# Physical Forcing Mechanisms Controlling the Variability of Chlorophyll-*a* over the Royal-Charlotte and Abrolhos Banks—Eastern Brazilian Shelf

**DOI:** 10.1371/journal.pone.0117082

**Published:** 2015-02-20

**Authors:** Renato David Ghisolfi, Meyre Pereira da Silva, Felipe Thomaz dos Santos, Ricardo Nogueira Servino, Mauro Cirano, Fabiano Lopes Thompson

**Affiliations:** 1 Oceanography and Ecology Department, Federal University of Espírito Santo, Vitória, ES, Brazil; 2 Rede de Modelagem e Observação Oceanográfica (REMO), Departamento de Física da Terra e do Meio Ambiente, Instituto de Física, Universidade Federal da Bahia, Salvador, Bahia, Brazil; 3 Biology Institute and SAGE-COPPE, Federal University of Rio de Janeiro, Rio de Janeiro, RJ, Brazil; University of Vigo, SPAIN

## Abstract

The Abrolhos Bank is part of the so-called Eastern Brazilian Shelf and is an area of high ecological and economic importance. The bank supports the largest and richest coral reefs in the South Atlantic and the largest rhodolith bed in the world. The spatial and seasonal variation of phytoplankton concentration, however, and the dynamic processes controlling that variability have remained poorly known. The present study investigates the seasonal and spatial distributions of chlorophyll-a (Chl-a) and water conditions by analyzing nine years (2003–2011) of level-3 Moderate-resolution Imaging Spectroradiometer (MODIS) derived Chl-a, National Centers for Environmental Prediction (NCEP)/ETA model-derived winds, NCEP model-derived heat fluxes, thermohaline and velocity results from the Hybrid Circulation Ocean Model (HYCOM) 1/12^o^ assimilated simulation. The results show that low/high concentrations occurred in austral spring-summer (wet season)/autumn-winter (dry season), with the highest values observed in the northern portion of the Abrolhos Bank. The typical meteorological and oceanographic conditions during austral summer favor the development of strong stratification. These conditions are 1) N-NE winds that favor an upwelling-type Ekman circulation; 2) coupling between the open ocean and the continental shelf through the western boundary current, which promotes cooler subsurface water to rise onto the shelf break; and 3) positive net heat flux. In contrast, the S-SE winds during autumn are in the opposite direction of the predominant current system over the Abrolhos Bank, thus reducing their speed and inducing an inverse shear. The warmer ocean and a somewhat cool and dry atmosphere promote the evaporative cooling of the surface layer. The above processes drive mixed layer cooling and deepening that reaches its maximum in winter. The blooming of phytoplankton in the Abrolhos Bank waters appears to be regulated by changes in the mixed layer depth, with Chl-a levels that start to increase during autumn and reach their peak in June-July.

## Introduction

**Fig 1:**
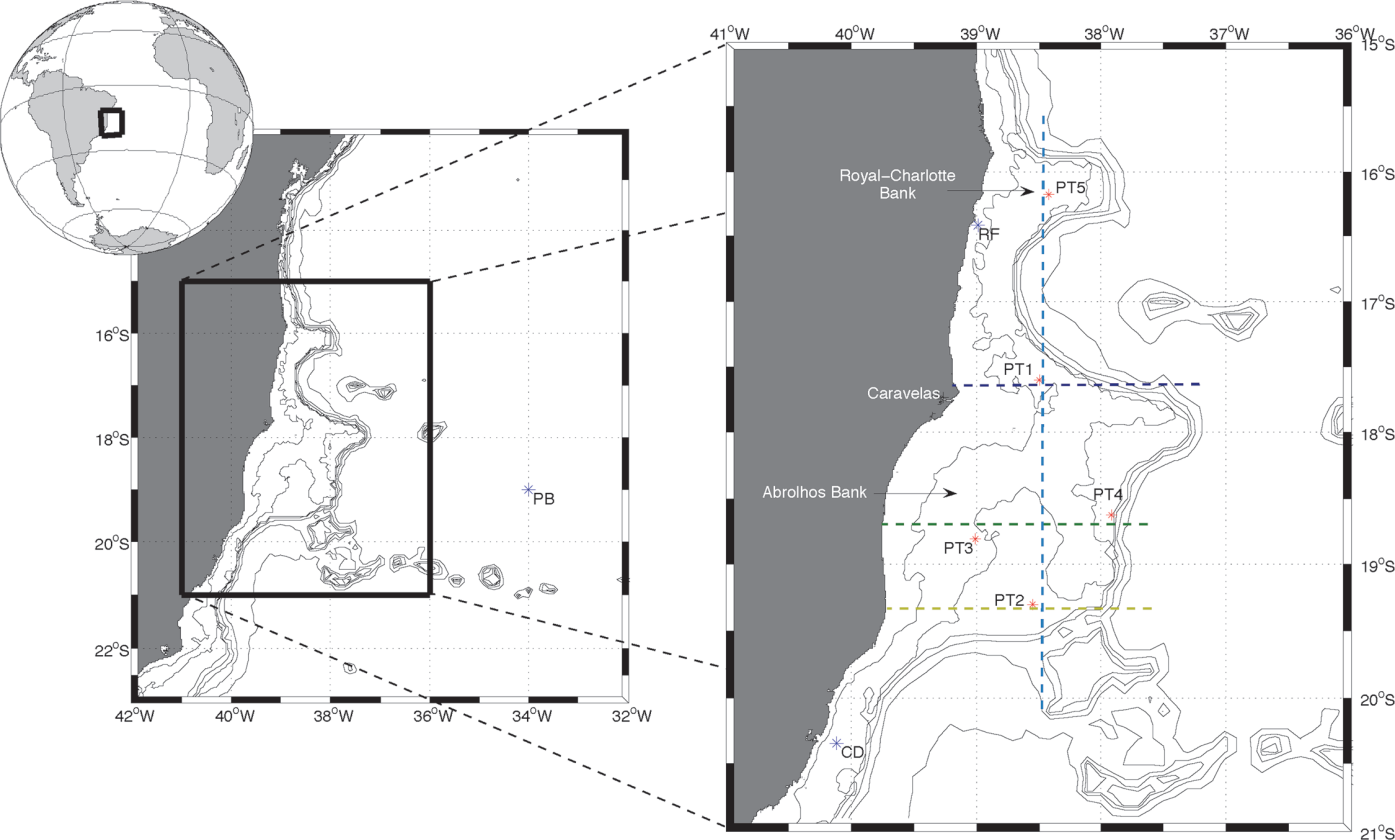
The study area showing the location of the five stations (PT1 to PT5) analyzed. Additionally, three cross-shore transects (R1—blue, R2—yellow and R4—green) and one alongshore transect (R3—cyan) were used to evaluate the seasonal variation of temperature in the water column throughout the study area. In situ wind data were obtained from a PIRATA buoy (PB in the left figure) and in situ current information was collected from a deployment designated by CD (figure on the right). RF stands for the Recife de Fora (Outer Reef). The isobaths shown are 25, 50, 200, 500, 1000 and 2000 m.

The Royal-Charlotte Bank (RCB) and the Abrolhos Bank (AB) are localized between 17°20’-18°10’S and 38°35’-39°20’W (Fig. 1) and comprise an extension of the Eastern Brazilian Continental Shelf (EBS) [1]. This section of the EBS is characterized by a wide continental shelf that extends almost 200 km in front of Caravelas (BA), as shown in Fig. 1. This region is an exception in an otherwise regular and narrow shelf. The northern part of the AB provides a home to the largest and richest reef system in the South Atlantic (Abrolhos reef system), which differs from the Caribbean reef system with respect to its reef morphology, reef building fauna and depositional setting [2]. The Abrolhos Shelf also harbors the largest continuous rhodolith bed in the world [3], which thrives in the outer and deeper parts of the Bank; the main reef-building corals, in contrast, are primarily restricted to the shallow areas (30–70 m depths).

Although phytoplankton biomass and turbidity have been frequently used as indicators of water quality in coral reef systems (e.g., [4]), there is little information on the seasonal and spatial distributions of chlorophyll-a (Chl-a) in the AB and RCB. The few existing oceanographic studies (e.g., [5], [6]) characterized this region as a transition zone between the low-productivity area found to the north [7] and the southern area in which coastal, shelf edge, and eddy-induced upwelling enhance the local productivity [8]. Mean Chl-a values of 1.22 mg m^-3^ (at the surface) and 0.86 mg m^-3^ (at the bottom) were reported by [9] for the inner reefs of Coroa Vermelha and Ponta Grande (just north and inshore of Recife de Fora—RF in Fig. 1). In contrast, the Chl-a concentration was approximately one third lower in both surface and bottom waters for the offshore reef of Recife de Fora (Fig. 1). Differences in Chl-a between nearshore and offshore reefs reflect the influence from terrestrial and nearshore sources of nutrients [9].

Due to the low riverine discharge, the most direct effects of land runoff are confined to the coastal area [10]. These effects present a significant seasonal variation in parameters such as nutrients and suspended material [11]. Under the influence of a tropical humid climate, the freshwater runoff is highest during the summer-wet season (December-April). Sediment resuspension forced by wind stress, primarily during winter storms, also affects the coastal reefs [12]. The Brazil Current (BC), which flows from lower to higher latitudes along the upper continental slope, intrudes over the AB, renewing it with clear and oligotrophic oceanic waters. According to [2], this process helps mitigate the land impacts on offshore reefs. To a lesser extent, wind-driven and tidal currents also contribute to inducing oligotrophic conditions over the AB [13].

Studies (e.g., [14], [15]) have reported that the coral reefs in the AB have been impacted by coastal eutrophication associated with sewage pollution. This problem, which is associated with the growing pressure of economic activities that have high potential for environmental impact (e.g., fishing, mining, oil and gas exploitation and dredging), has created a demand for conservation and for broader management strategies of AB marine resources [16]. However, to implement any conservation plan or management strategy, it is first necessary to improve the understanding of the oceanographic and land-driven influences on the AB and RCB ecosystem.

Changes in phytoplankton biomass and productivity at seasonal to inter-annual time scales are very important components of the total variability associated with ocean biological and biogeochemical processes [17]. Furthermore, understanding the spatial and temporal patterns in phytoplankton distribution can provide important information about fluctuations in coral reef health [18]. Though a significant understanding of the circulation of the AB was developed using ocean models over the last decade, we still know little about how that circulation relates to biological processes in the water column. This lack of information is primarily due to the lack of comprehensive observational data. Most of the available oceanographic and biological data are restricted in both space and time and are thus not suitable to study patterns over the AB and RCB.

Few studies have explicitly investigated the relationship between the meteorological and oceanographic conditions and the phytoplankton dynamics. Most such studies have associated the wintertime increase in Chl-a concentration with the wind regime and associated turbulence (e.g., [19], [20]). However, other factors controlling mixed layer dynamics such as heat fluxes have not been considered. In this study, a combination of satellite-derived Chl-a data and numerical simulation results was analyzed to investigate the patterns and mechanisms behind phytoplankton variability in the AB and RCB regions. From these data, a better understanding of the link between changes in meteorological and oceanographic parameters and phytoplankton dynamics can be reached. This article is organized as follows: the data and methods are presented in section 2, and the results are described in section 3. A discussion of the results is presented in Section 4, and the conclusions are drawn in the final section.

## Data and Methods

Time series of satellite-derived Chl-a were constructed for five locations (one over the RCB and four over the AB—Fig. 1) from nine years (2003 to 2011) of 8-day composite Chl-a imagery acquired from MODIS level 3 products (freely available from *oceancolor*.*gsfc*.*nasa*.*gov*). Their locations were chosen based not only on the spatial representativeness of the study area but also on the spatial-temporal variability observed in the mean seasonal Chl-a images. Points PT1 and PT3 are located where the benthos is covered by coral reefs, and points PT2, PT4 and PT5 are located in rhodolith-covered benthic regions [16]. Missing data were filled in using a mean climatological value for the same point and represented less than 30% of the dataset for each location.

Bio-optical and atmospheric correction algorithms are important components in processing satellite ocean color data. Most bio-optical algorithms have been empirically derived ([21], [22]) and quantify the Chl-a concentration as a function of the blue-to-green reflectance ratio [23]. These algorithms perform well in Case 1 waters but exhibit limitations in optically complex waters in which optically active components other than chlorophyll are present. The comparison between in situ and satellite-derived Chl-a has been used to ensure that satellite-derived measurements are representative of the true Chl-a concentrations; several studies have evaluated the performance of the standard MODIS algorithms for locations worldwide (e.g., [24], [25], [26]). However, there are no validation studies of MODIS-derived Chl-a concentration in the area of the present study, so the data presented here should be considered primarily in terms of their variability rather than their absolute values. Nevertheless, the in situ Chl-a results reported by [27] for January 2009 and 2010 for the AB (0.17 to 0.50 mg m^-3^) were in the range of those found in this study.

To investigate and quantify the phenology of the seasonal bloom in tropical latitudes, the 8-day Chl-a values were averaged monthly over the 9-year period and fitted to a Gaussian function [28] of Chl-a versus time according to the methodology described in [29], [30], [31] as

$$Chl(t)=Chl_{o}+\frac{h}{\sigma \sqrt{2\pi }}\exp\left[-\frac{{\left(t-t_{\max}\right)}^{2}}{2\sigma^{2}}\right]$$ Equation1

where Chl_o_ is the baseline of Chl-a concentration (mg m^-3^), t_max_ (month) is the time at the peak concentration and σ (month) is the standard deviation of the Gaussian curve. The peak concentration is defined by ($h/\sigma \sqrt{2\pi }$), where h (mg month m^-3^) is the integral of all satellite-derived Chl-a values above the baseline. This formulation was also used by [32], [33] and [30]. The initiation of the bloom was estimated as the time when the Chl-a concentration was 10% above the annual median value, similar to the definition used by [34]. The same criterion was used to define the end of the bloom in the downslope of the Gaussian curve. The time interval between the beginning and end of the bloom was assigned as the duration of the bloom (month).

Due to the lack of in situ data with adequate spatial and temporal resolution to examine the physical forcing that controls the distribution of Chl-a in the AB and RCB region, this analysis uses a combination of data from a satellite and a heterogeneous set of modelling results. Daily 3-D temperature, salinity, velocity and the Sea Surface Height (SSH) outputs from Hybrid Ocean Model (HYCOM [35]) global assimilated simulations were downloaded freely from the *hycom*.*org* website. The data are available at standard oceanographic depths with a spatial horizontal resolution of 1/12° from January 2004 up to December 2011. The meridional and zonal components of the wind velocity (0.2° spatial resolution) were obtained from the ETA model [36], [37] through the Instituto Nacional de Pesquisas Espaciais (INPE-Br), covering the period between May 2005 and December 2010. Though the datasets do not cover the entire 9-year period of Chl-a data, the information available was determined to be suitable for this study.

To validate the model results, a comparison with available observations was performed. The representativeness of the simulated wind values was tested against in situ information collected at the PIRATA buoy (PB—Fig. 1). The hydrodynamics were compared to in situ data obtained from a 5-month shallow water deployment (CD—Fig. 1). Although the in situ observations were not collected within the study area, the comparison was performed to include at least an evaluation of the model results at the closest region available. These analyses were based on a Taylor diagram (Figures not shown); for the wind data, the correlations coefficients were higher than 0.7 (root mean square difference for the X component—rmsd_x = 2.71 m s^-1^, and Y component—rmsd_y = 2.63 m s^-1^, p<0.05) and were just over 0.5 for the U (rmsd = 0.20 m s^-1^, p<0.05) and V (rmsd = 0.17 m s^-1^, p<0.05) current velocity components.

The skill for temperature and salinity in a HYCOM assimilation were evaluated using 2 years (2010–2011) of temperature and salinity observations from 16 autonomous underwater glider vehicle missions and four hydrographic voyages in the Mid-Atlantic Bight [38]. The results indicated that, in general, HYCOM is biased warm and slightly salty. The skill is greater near the surface and decreases with depth, reaching a minimum at mid-depth (25–50 m) in summer, but decreases steadily with depth, without a local minimum in winter. On the EBS, the HYCOM assimilation was compared with a set of thermal records collected by 4 iButton sensors that were deployed for 1 year (June of 2006 to May of 2007) on the bottom (54 m) on the outer shelf, at approximately 21°03’ S and 40°18’ W. Similar to the results found in the Mid-Atlantic Bight, the temperature and thermal seasonal cycle is more pronounced in the HYCOM results than in the observations. The correlation coefficient between the averaged time series from the 4 temperature sensors and the temperature time series from the grid point closest to the deployment is 0.4 (p<0.01) and increases to 0.5 (p<0.01) when comparing temperature anomalies (monthly mean removed).

Despite the relatively low correlations between the HYCOM results (velocities and temperature) and the observations, the datasets were considered adequate given the spatial and temporal interpolation required for the comparison.

To identify the periodic components of the time series, a Fast Fourier Transform (FFT) was applied to all detrended series available using their original temporal resolution [39]. However, the datasets were averaged to the lowest temporal resolution to estimate the correlation between the time series and coherence between spectrums when FFT was then reapplied. Finally, unless stated otherwise, the first, second, third and fourth trimesters of the year represent summer, autumn, winter and spring, respectively.

## Results

### Chl-a distribution

**Fig 2:**
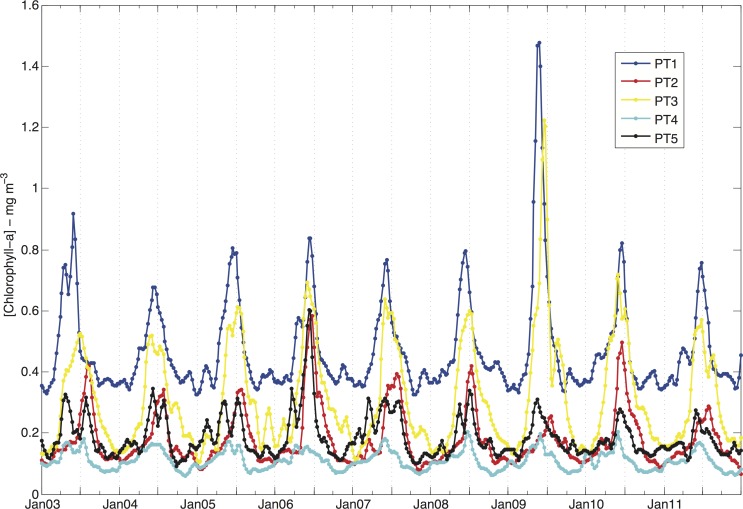
Chl-a distribution between 2003 and 2011 at the five locations indicated in Fig. 1. A running mean of 3 points was applied to all series. The unlabeled tickmarks on the x-axis represent the months of June (major tickmarks) and April and September (minor tickmarks).

In the five time series shown in the Fig. 2, higher concentrations were observed in the end of autumn and beginning of winter (June/July), whereas the Chl-a values were lower in spring and summer. The values ranged from 0.1 mg m^-3^ to 1.5 mg m^-3^, with lower values (< 0.3 mg m^-3^) at points PT2, PT4 and PT5 and relatively high concentrations (> 0.4 mg m^-3^) at the station located in the Abrolhos reef area (PT1). The largest difference between the minimum and maximum values was observed at point PT3. This apparent spatial dependence of the Chl-a distribution was confirmed by a non-parametric statistical analysis (Kruskal-Wallis test, p<0.05–[40]). According to the Kruskal-Wallis test, two clusters were obtained: Group I, which included points PT1, PT3 and PT5, and Group II which contained points PT2 and PT4. The former gathered points located close to the coast and in the northern part of the bank, whereas the latter included points located in the southern and eastern parts of the AB. This test also revealed significant Chl-a differences among months within each point but detected no interannual variability for the 9-year period analyzed.

The periodograms associated with Chl-a distributions (not shown) indicated that most of their energy variance was related to the annual cycle. Although the semi-annual and higher frequency peaks were also present, the energy associated with higher frequencies was at least three-fold lower. The characteristics of the seasonal bloom for each point were evaluated using a Gaussian function (given by Equation 1) fitted to the mean monthly Chl-a time series. High values of the coefficient of determination (see Table 1 for the complete set of parameters) were found at all sites. Using the average of the parameter values for all 5 points, the mean bloom started in approximately mid-March, reached its peak in June and lasted approximately 5 months.

**Table 1:** Parameters obtained from the Gaussian fit for the seasonal climatological bloom: the baseline chlorophyll concentration (Chl_o_), the maximum amplitude (Peak), the time of initiation (T_start_) and duration of the bloom.

Point	Chl_o_ (mg m^-3^)	Peak (mg m^-3^)	T_start_ bloom (month)	T_max_ bloom (month)	Duration of the bloom (month)	R^2^
1	0.3786	0.7937	3.56	5.77	4.50	0.97
2	0.1156	0.3354	4.19	6.81	5.25	0.97
3	0.1686	0.6035	3.46	6.29	5.64	0.96
4	0.0797	0.1598	2.62	5.40	5.64	0.88
5	0.1398	0.2884	3.40	5.83	4.88	0.94

R^2^ is the determination coefficient of the Gaussian fit. The months are indicated by numbers from 1 (January) to 12 (December).

The parameter values for each point revealed spatial differences in phytoplankton phenology, suggesting two distinct ways of grouping the points. The first one was based on the timing of the bloom initiation and the peak: for the point farthest from shore (PT4), the bloom started and peaked earlier than the average, late February and mid-May, respectively; for the midshelf points (PT1, PT3 and PT5), the values were closer to the average; and for the point situated at the southern end of the AB (PT2), the bloom started (beginning of April) and peaked (end of June) later than the average. This pattern resembles the cluster suggested in the Kruskal-Wallis analysis, except the points PT2 and PT4 were grouped together in the test’s cluster Group II but here represent opposite extremes. In terms of the duration of the bloom, the points were grouped differently: at point PT1, the duration was shorter than average (4.5 months), closer to the average at points PT2 and PT5 and longer than the average (5.6 months) at points PT3 and PT4.

To decipher the coupling of chlorophyll-a with physical parameters and understand how these parameters control the spatial structure of the seasonal bloom, the seasonal pattern of mixed layer depth, wind, water temperature, surface fluxes, Ekman pumping and SSH are examined next.

### Meteorological and oceanographic forcing

FFT analysis was applied to time series of water temperature, currents and wind. Regardless of the variable, the highest energy was associated with the annual cycle.

**Fig 3:**
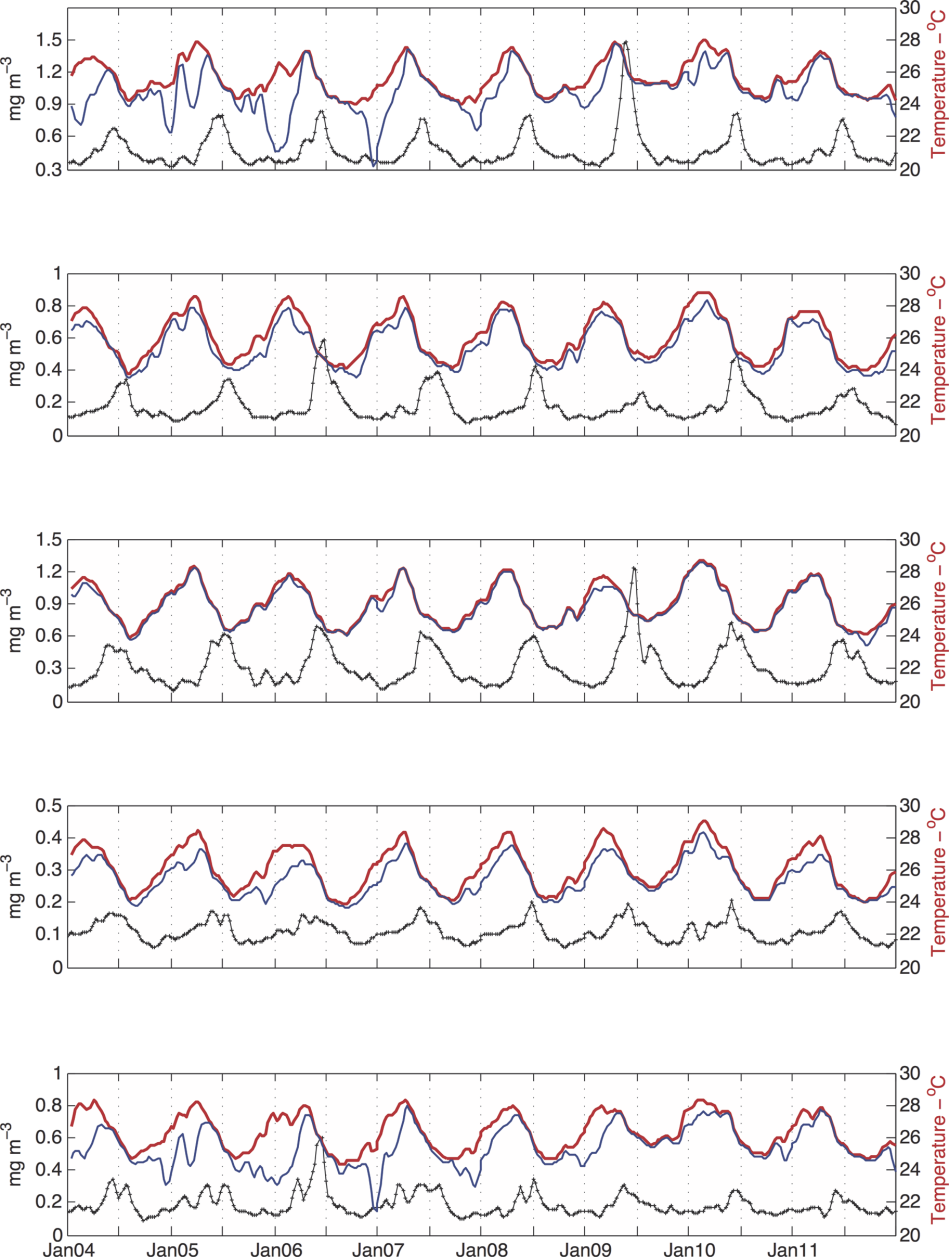
Temperature distribution at the surface (red), 30 m depth (blue) and the Chl-a concentration (black) for points PT1 (top) to PT5 (bottom). Every temperature value corresponds to the mean for the same 8-day periods as for the level 3 processed Chl-a data. A 3-point running mean filter smooths the curves. The unlabeled x-tickmarks represent July of the corresponding year.

The seasonal heating (September to February) and cooling (March to August) cycle in the upper layers on the banks is evident in Fig. 3. As a general pattern, the temperature reached maximum values from the middle to end of summer. Conversely, minimum values occurred from the end of winter to beginning of spring. With the exception of PT3, where the water column was thermally homogeneous throughout the year, a more pronounced vertical thermal gradient was clearly observed during the heating period for all points. The differences in temperature between the surface and 30 m depth were greatest in January (austral summer), as opposed to July (austral winter), when the temperature differences between these two depths were almost undetectable. At points PT1, PT4 and PT5, the vertical stratification of the water column seemed related not only to the seasonal heating but also to the intrusion of colder waters onto the bottom of the continental shelf.

**Fig 4:**
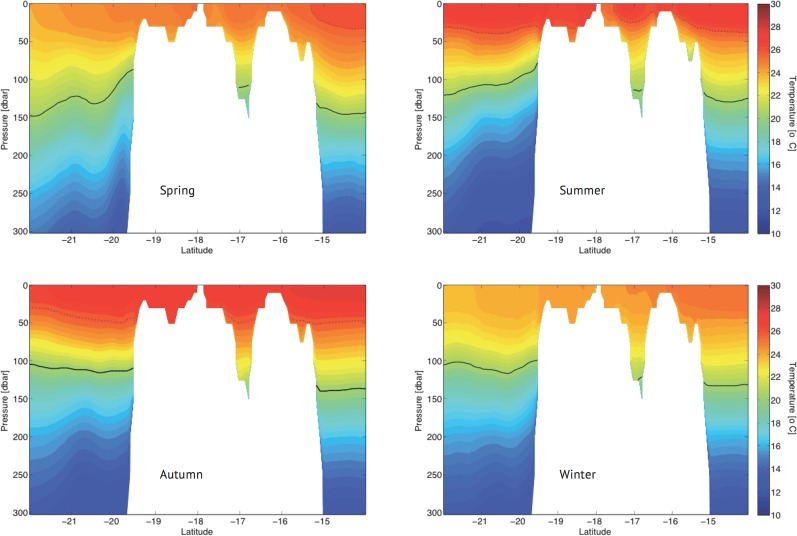
Seasonal vertical temperature field in Radial R3 for the year 2008. The dashed line indicates the 26° C isotherm and the solid line is the upper temperature limit of SACW, which was always below 100 m depths.

An alongshore (R3) and a cross-shore (R4) transect (Fig. 1) were chosen for a more detailed examination of the seasonal variability of the vertical temperature distribution at the selected points. The alongshore transect (Fig. 4) shows that during spring (top left panel), water warmer than 26°C was restricted to the northern part of the region, whereas during the summer, it was distributed throughout the surface region (top right panel). The inclination of the isotherms suggested the presence of colder water on both sides of the embayment of Royal-Charlotte-Abrolhos (latitude 17°00’S) and on the east side of the RCB. Evidence of anti-cyclonic eddies (Royal-Charlotte and Ilhéus, respectively to the left and right of RCB) have been reported by [41]. These features may be responsible for the observed uplift because water could sink in the middle of these eddies and upwell along their peripheries, particularly when such anti-cyclonic eddies approach the shelf border. During autumn and winter (bottom left and right panels, respectively), the first 50 m were thermally homogeneous, warmer in the former and relatively colder in the latter. These results confirmed that the vertical temperature distributions at points PT1 (17°48’S) and PT5 (16°36’S) were influenced by upwelling primarily in summer. In contrast, point PT2 (19°24’S) was located in a more sheltered position and was thus not exposed to the action of the BC eddies and meanders.

**Fig 5:**
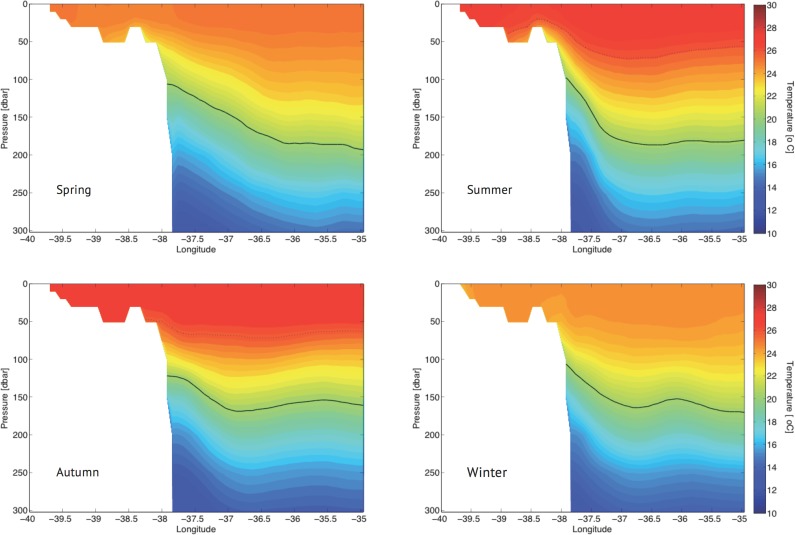
Seasonal vertical temperature field in Radial R4 for the year 2008. The dashed line indicates the 26° C isotherm and the solid line indicates the upper temperature limit of SACW, which was always below 100 m depths.

The vertical temperature distribution at points PT3 and PT4 is better visualized from the cross-shore transects R4 (Fig. 5). The uplift of the isotherms along the shelf break was evident in spring (top left panel) and even more notably in summer (top right panel). During these periods the Abrolhos Eddy [41] situated close to the coast might have driven upper thermocline waters onto the shelf. In autumn (bottom left panel), the mixed layer was fully developed over the continental shelf, whereas a weak stratification developed in the shelf break area in winter (bottom right panel), possibly driven by the Abrolhos Eddy. The vertical temperature distribution at PT4 (37°45’W) was directly influenced by open ocean dynamics, similar to points PT1 and PT5. Because of its location closer to the coast, point PT3 (39°00’W) was not affected by shelfbreak upwelling. This site therefore developed relatively weak vertical temperature stratification, even during summer, as described above. Although not shown, Radials R1 and R2 presented a similar pattern as described for Radial R4.

**Fig 6:**
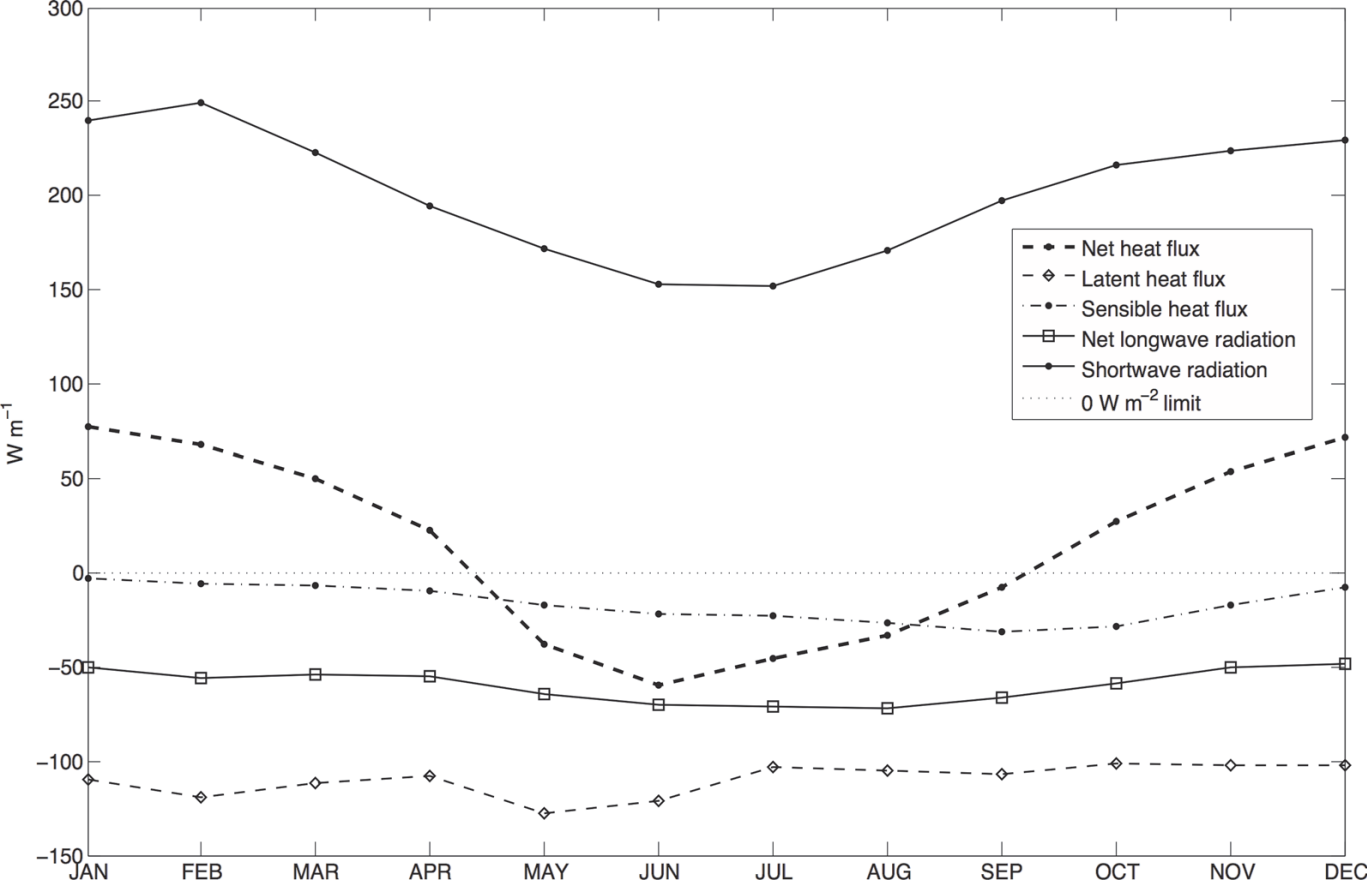
Monthly mean components of the heat flux balance across the air-sea interface, valid for the Abrolhos region and estimated from NCEP reanalysis data. The black dashed line represents the net heat flux. When the ocean loses heat, the net heat flux is negative.

The highest Chl-a concentration occurred when the temperature was decreasing (at PT1, PT4 and PT5) or had reached its minimum (at PT2 and PT3). A closer inspection of Fig. 3 reveals that the peaks of Chl-a were also associated with thermal homogeneity in the water column (the thickening of the mixed layer). The deepening of the mixed layer is regulated by factors such as mixing associated with negative surface buoyancy flux due to cooling of the surface waters and/or the downward transfer of momentum by the wind stress. Fig. 6 shows the mean monthly net heat flux balance across the air-sea interface for the area. The heating-cooling cycle varied according to the shortwave radiation income. Broadly speaking, from October to April, when the shortwave radiation is greater than 200 W m^-2^, the surface ocean warms up, which favors a reduction in the mixed layer thickness and the development of a seasonal thermocline [42], [43].

The seawater over the banks loses heat from mid-April to September. The loss is conditioned mainly by the reduction in shortwave radiation income and both a slight increase in latent heat loss from April to July and a net longwave heat flux from April to October. It has been suggested that the meridional and zonal displacement of the anti-cyclone high-pressure system (AHPS) as shown in [44], controls the most important ocean heat loss fluxes. During summer, the center of the AHPS is situated near the African coast. The atmospheric pressure gradient between the ocean and the South American continent is high, as are the winds in the Abrolhos region. With the onset of autumn, the pressure system center starts to elongate in the east-west direction and moves westward, and the pressure maximum then increases. However, the pressure gradient between ocean and continent decreases. Because it is a high-pressure system, dry air moves downward and spreads out in the anti-cyclonic sense. Dry air blowing over a warm ocean increases the loss of latent heat because of the high difference in humidity rather than an association with increase in wind speed. A less humid atmosphere favors the loss of longwave radiation because there is no water vapor in the atmosphere to block it and re-emit it downward. Considering the surface fluxes, the cooling of surface waters promoted vertical convection and thus the mixing and thickening of the mixed layer.

**Fig 7:**
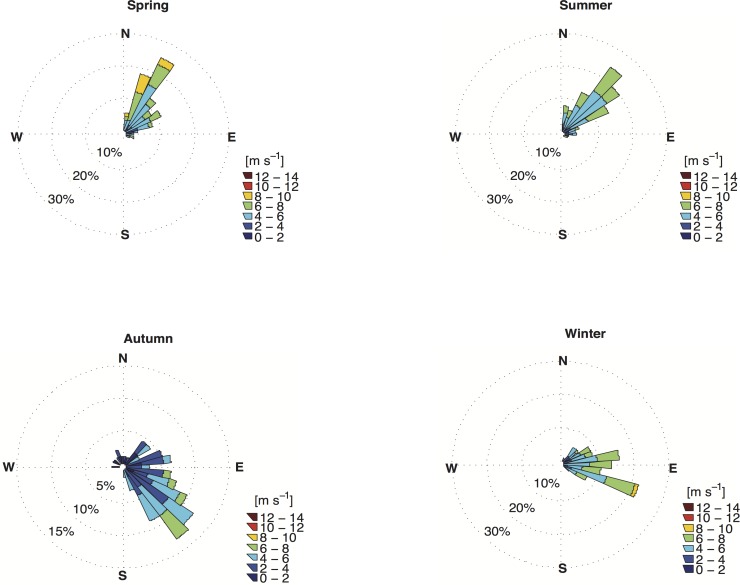
Seasonal wind rose climatology for the Abrolhos area for the period 2005–2010. Stronger (NE)/weaker (SE) winds occurred in spring/autumn, respectively.

**Fig 8:**
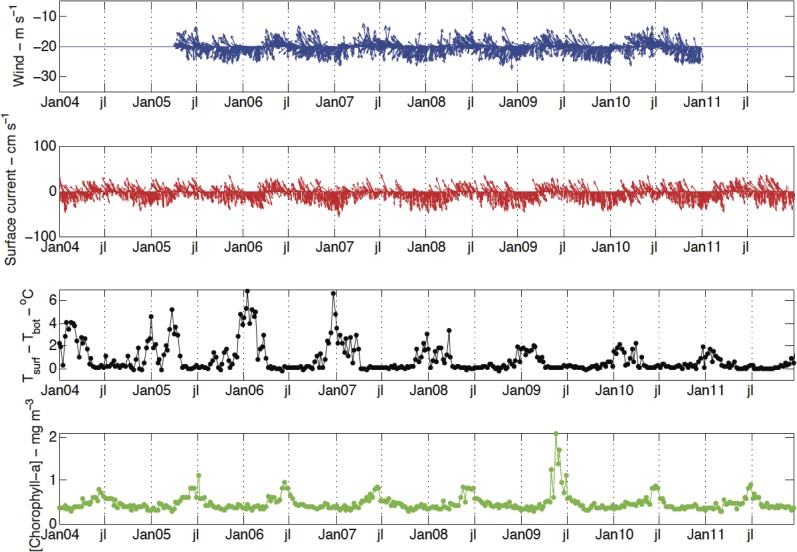
Temporal distribution of wind (blue), surface currents (red), the temperature difference (T_surf_-T_bottom_) (black) and Chl-a concentration (green) at point PT1. July is abbreviated as jl. The wind direction follows the oceanographic notation convention.

The input of momentum into the mixed layer by wind stress should create shear-driven instability and vertical mixing. Thus, it would be expected for stronger winds to be associated with a deeper mixed layer. The seasonal wind climatology based on the available data (Fig. 7) revealed that relatively stronger northeast winds were typical during spring and summer, whereas the onset of autumn corresponded to relatively weak southeast winds that later strengthened slightly; easterlies were typical in winter. When associating the results in Fig. 7 with those shown in Figs. 3, 5 and 6, the outcome is the opposite of that expected (Fig. 8). For the sake of simplicity, the result is shown only for point PT1. Stronger north-northeast winds resulted in higher vertical thermal gradients, south-southwest currents [45] and lower Chl-a concentrations. In contrast, south (southeast-east) winds were linked to essentially barotropic north (northeast) surface currents [45] (not shown), smaller thermal gradients and higher Chl-a concentrations. Therefore, as in shallow tropical areas [46], the development of the winter mixed layer in the RCB and AB is driven mainly by the net heat flux.

**Fig 9:**
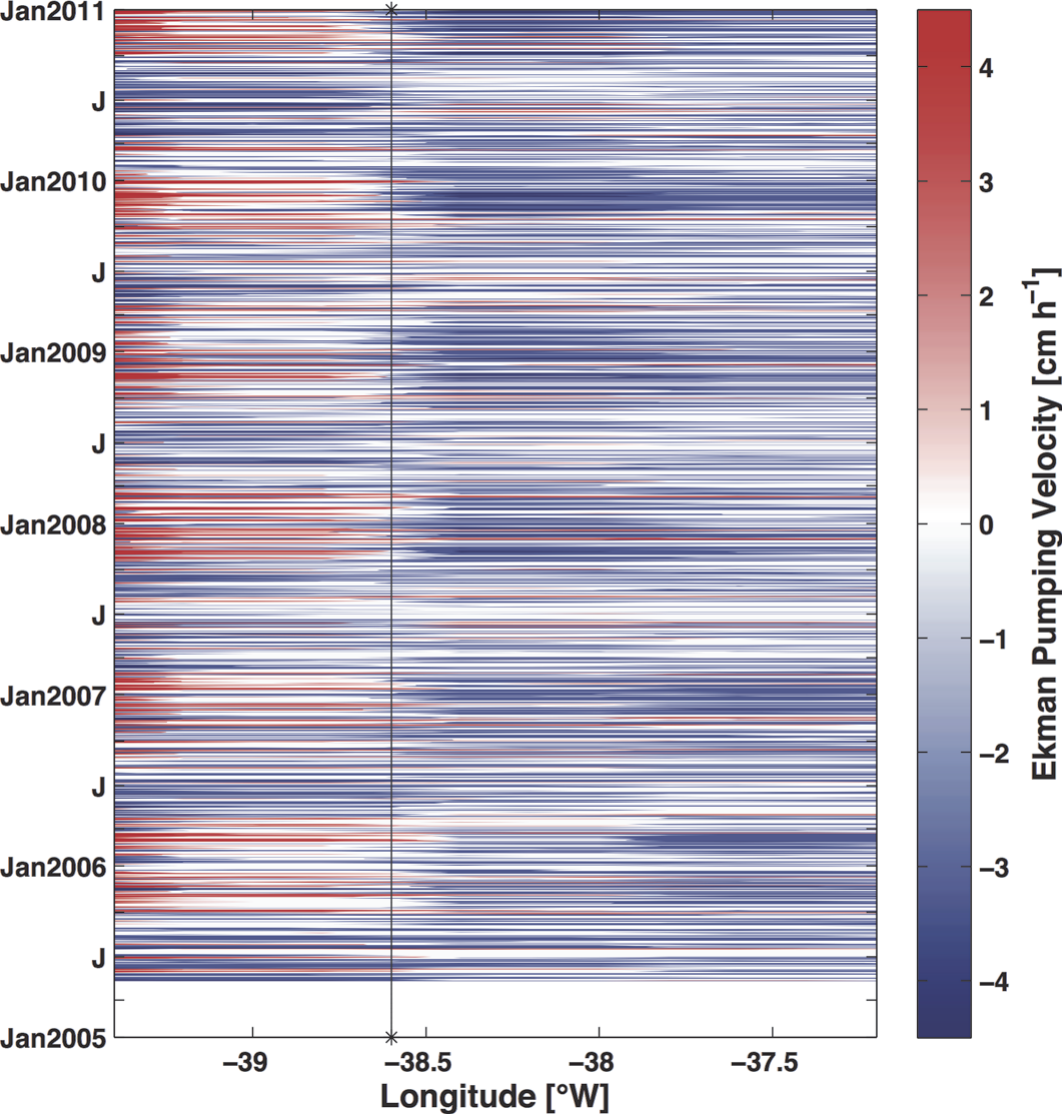
Temporal-spatial distribution of Ekman pumping velocity over the Abrolhos Bank along a section passing through point PT1. The black line indicates the longitudinal position of point PT1.

The time-longitude distribution of the Ekman pumping velocity was evaluated in 5 zonal sections corresponding to the locations of the 5 points. Fig. 9 shows only the result from the section passing through point PT1 because the remaining four transects showed a similar pattern. The temporal variability of the other 4 points can be inferred from their position in relation to point PT1 (Fig. 1). During summer, upward velocities reaching more than 3 cm h^-1^ occurred in the inner shelf (onshore of PT1 location) (Fig. 9), whereas in the rest of the bank, the velocities were downward. In the winter periods (centered on July), downward velocities instead tended to occur along the entire bank. Therefore, Ekman pumping alternated between upward and downward velocities during the year in the inner shelf. In the middle and outer shelf, however, downward velocities prevailed throughout the year. The SSH results from the HYCOM simulation (not shown) corroborate these findings.

**Fig 10:**
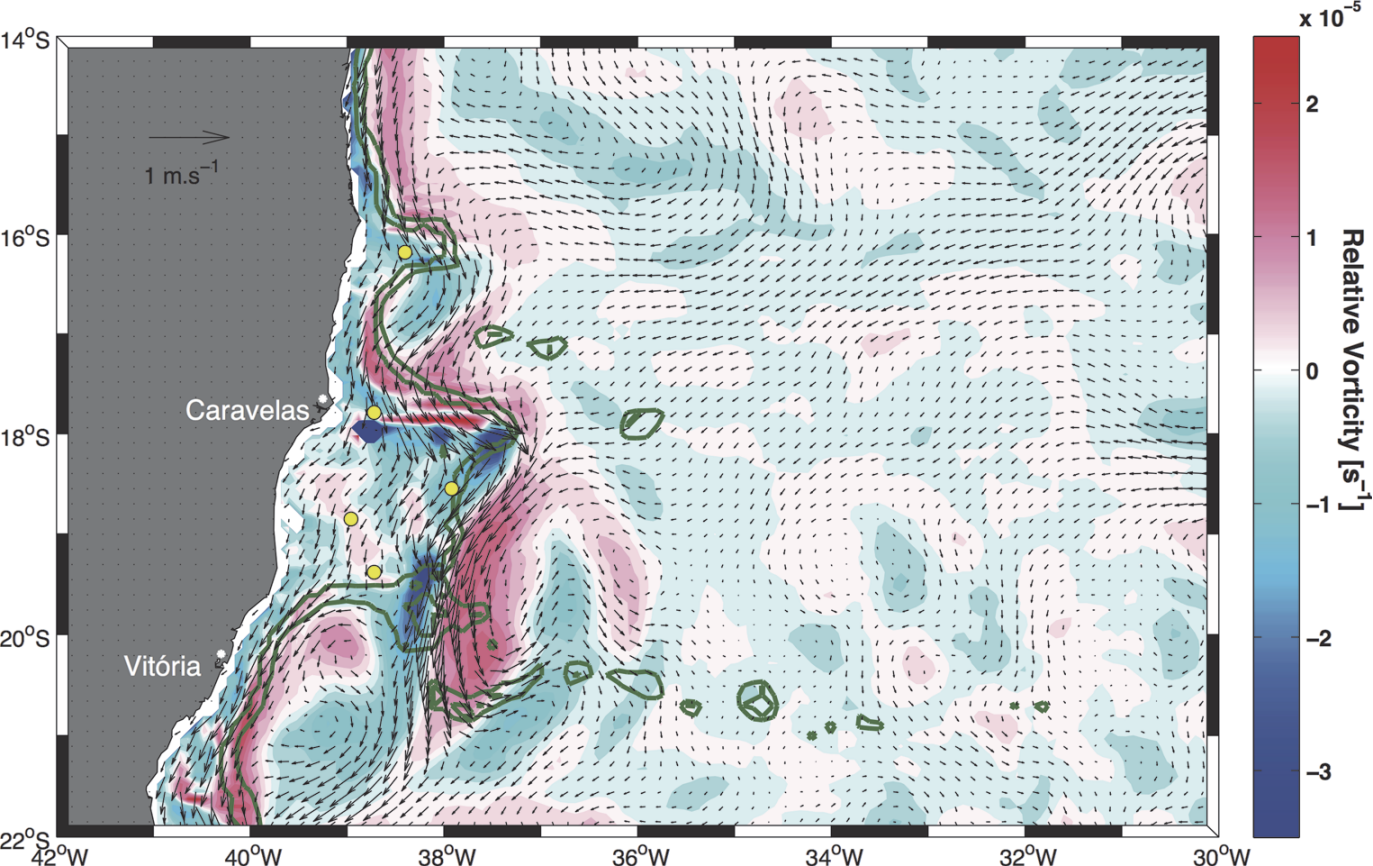
Seasonal surface relative vorticity field computed from the mean summer current field overlap. Colors indicate the magnitude of relative vorticity. Yellow circles mark the position of the five points indicated in Fig. 1. Green lines indicate the 200 m and 1000 m isobaths.

**Fig 11:**
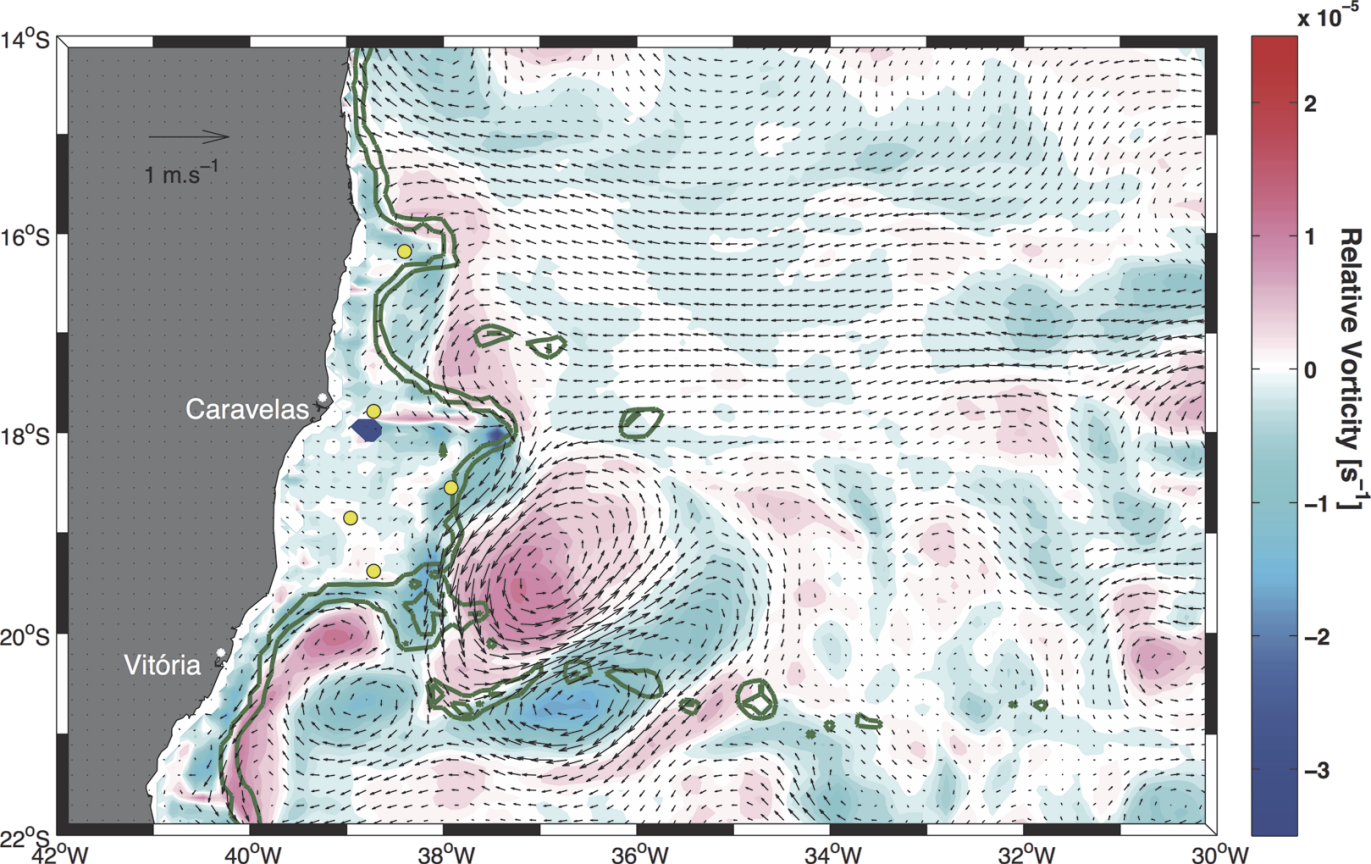
Seasonal surface relative vorticity field computed from the mean autumn current field overlap. Colors indicate the magnitude of relative vorticity. Yellow circles mark the position of the five points indicated in Fig. 1. Green lines indicate the 200 m and 1000 m isobaths.

The influence of relative vorticity on the enhancement or weakening of the thermal upper layer gradient was estimated by computing the surface relative vorticity fields based on the mean seasonal surface current fields. The results revealed that in summer, higher positive (negative) values of the vertical component of relative vorticity ζ_z_ were associated with the inner (outer) portion of the BC jet (Fig. 10), a feature that can easily be identified as a well-organized flow present along the shelf break [47]. In fact, relative vorticity was mainly negative (upward) at points PT1, PT4 and PT5, whereas it was slightly positive (downward) at points PT2 and PT3. During autumn (Fig. 11) and winter, the relative vorticity field was weak because of a weak hydrodynamic field. The strong BC flow in the previous season (Fig. 10) was then reduced to a weak flow along the shelf break region. Nonetheless, the approximately null velocity field over the banks shown in Fig. 11 is a deceptive feature. Rather than indicating that water on the continental shelf region was stagnant, it indicated that surface currents were highly variable [48] and that the mean seasonal value was near zero.

## Discussion

Regardless of some energy associated with the semi-annual cycle, the significant strong annual signal of Chl-a [29] identified in all other time series analyzed is the focus of this discussion. Therefore, unless discussing the results in terms of the four seasons of the year, the results were framed in terms of the Köppen climate classification [49]. As stated by that author, the region where the AB and RCB are located has a pseudo-equatorial climate with a wet season (from October to March—spring and summer) and a dry season (from April to September—autumn and winter).

As a general feature, low (high) concentrations of Chl-a were associated with the wet (dry) season. This result has been reported in previous studies performed in the South Atlantic Bight (e.g., [50], [8], [51]), southwestern tropical Pacific (e.g., [52]) or in Brazilian waters (e.g., [53], [54], [19], [55], [20]); the increase in the Chl-a concentration during winter months was associated with the fertilization of surface layers as a result of vertical mixing. These results indicated that, as in other tropical regions, the key factor limiting phytoplankton growth is likely to be the accessibility of nutrients rather than light.

Previous studies (e.g., [54], [19]) have associated the nutrient enrichment of the euphotic zone with stronger southerly winds that would erode the thermocline, thus fertilizing the surface layers. For the region considered in this study, this cause-effect relationship (that is, stronger winds being positively correlated to Chl-a concentration) does not seem as clear. The correlation between Chl-a and wind intensity was not significant for points PT1 and PT5; while for points PT2, PT3 and PT4, weak negative correlations between those variables were found (mean correlation coefficient—r_ave_ = -0.22, p<0.05). In contrast, a negative correlation (r_ave_ = -0.46, p<0.0001) between Chl-a and the meridional wind component was identified; high Chl-a concentrations were associated with relatively weak southerly winds. Similar negative correlations (r_ave_ = -0.38, p<0.005) were obtained between the mixed layer depth (depth at which the surface **σ**_**t**_ was 0.125 kg m^-3^ greater—[56]) and the meridional wind component, associating a deeper mixed layer with weak southerly winds.

**Fig 12:**
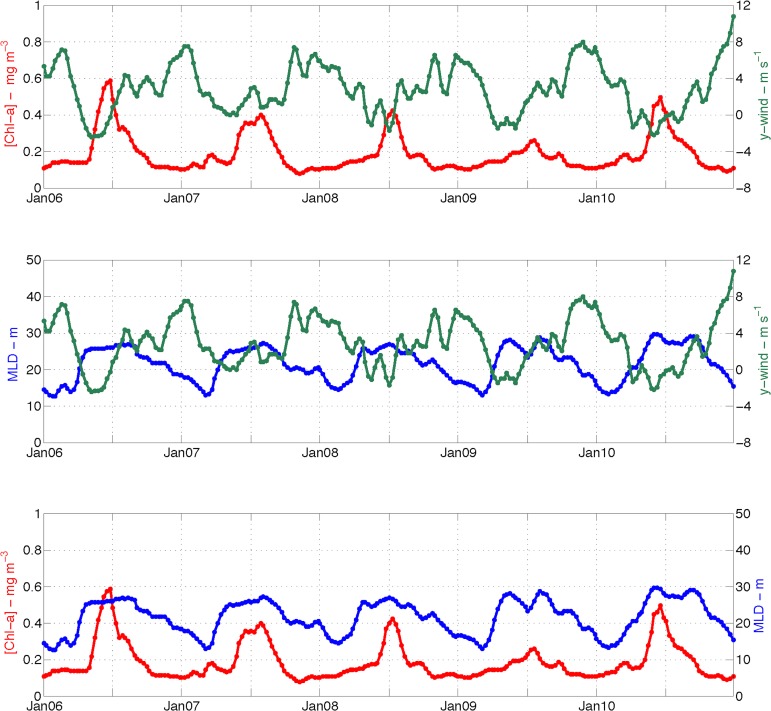
Chl-a (red), MLD (blue) and the meridional wind component (green) between 2006 and 2010 at point PT2. Every point corresponds to a mean of the same 8-day values as for the level 3 processed Chl-a data. A 3-point running mean filter smooths the curves. Unlabeled x-tickmarks represent July of the corresponding year.

Southerly winds were a common feature of the dry season, as shown in the results section (Fig. 7), but these weak winds occurred in the study area after the mixed layer had begun to deepen or had reached its maximum depth (e.g., point PT2—Fig. 12). Furthermore, stronger northerly and northeasterly winds were typical in the wet season and were associated with shallower mixed layers and vertical temperature stratification (Fig. 3). Therefore, the idea often assumed in tropical and subtropical regions (e.g., [57]), i.e., that stronger winds can increase the upward flux of nutrients by increasing vertical mixing and deepening the mixed layer, cannot explain the seasonal bloom shown in this study. However, it should not be discounted that a short-term variability in wind direction and intensity may drive higher frequency fluctuations in the mixed layer depth and Chl-a concentration.

The results shown in Figs. 3 and 8 indicate a positive association between high levels of Chl-a to vertical temperature homogeneity. Indeed, Chl-a showed a moderate to weak correlation with mixed layer depth (r_ave_ = 0.33, p<0.05). PT2 displayed the highest correlation (r = 0.5, p<0.00001); the correlation value at PT4 was just above the significance level (r = 0.16, p<0.05). Intermediary correlation values of approximately r = 0.3 (p<0.00001) were found at PT1, PT3 and PT5. The coupling between Chl-a and mixed layer depth separated the points into 3 groups in a fashion similar to that based on the timing of the initiation of the bloom. This similarity suggests that the spatial variation in the bloom timing over the AB and RCB was to some extent controlled by mixed layer depth.

At points PT1, PT4 and PT5, the seasonal bloom started when the mixed layer depth began to increase but when the mixed layer reached the maximum depth for points PT2 and PT3. With the exception of point PT4, the bloom initiation coincided with the onset of the dry season (Table 1) when the ocean surface started losing heat (Fig. 6). A negative correlation was therefore expected between Chl-a and the seasonal mean of the net heat flux. The correlation coefficient values ranged from r = -0.64 (p<0.05) at point PT4 to r = -0.96 (p<0.00001) at point PT3. A negative correlation is the opposite of that expected for polar and sub-polar latitudes ([50], [58]). In those regions, the phytoplankton vernal bloom occurs when the water column stratifies due to an increase of insolation and reduction of winter mechanical forces; these changes reduce the mixed layer depth and trap phytoplankton above the critical depth [59]. Here, heat flux drove the deepening of the mixed layer. A reduction in the photoinhibition of plankton [60] is then likely to occur due to the vertical displacement of planktonic cells and to the vertical mixing that allowed nutrient rich bottom waters to reach surface layers and introduce nutrients into the photic zone [51], thus favoring an increase in Chl-a concentration.

The present results suggest that the seasonal thickening of the mixed layer associated with a heat loss-driven convection, rather than wind, may be the major factor controlling Chl-a seasonal blooms over the AB and RCB. However, the weak correlation between Chl-a and mixed layer depth estimated for some points (e.g., PT4) suggests that the ocean dynamics may play a role in conditioning the bloom [48]. Upwelling of sub-mixed-layer waters onto the continental shelf promoted by eddies and meanders associated with the BC, upwelling favorable wind stress, current-driven processes and Ekman pumping [61], might have triggered the earlier start of the bloom at point PT4. These environmental changes may also be reflected in the weak coupling between Chl-a and mixed layer depth. Although this process is more intense and frequent during the wet season, it can also occur during the dry season. Using SST data from the NOAA Pathfinder Program, [62] verified that during the summer, the SST over the northern portion of AB (PT1) and RCB (PT5) were 1° C colder than their surroundings. During winter, this feature is also present but is less pronounced, most likely due to upper layer cooling.

The contribution of each of the mechanisms cited above for the uplift of cooler and most likely nutrient enriched water remains unclear, as do the biological implications of such uplift. However, it is possible that the nutrient enriched water brought up onto the banks does not result immediately in higher levels of Chl-a. The vertical stratification due to the surface insolation and the presence of colder water at the bottom may inhibit this process. The result would be a shallow mixed layer that is unfavorable to the supply of nutrients through entrainment processes to the euphotic zone. Furthermore, low Chl-a concentrations in the wet season may be due to the efficient flushing promoted by the BC, flowing over the banks washes off nutrients, leaving oligotrophic conditions behind [63].

During the dry season, the wind direction rather than its intensity may play a role in maintaining the water column mixing (Figs. 4 and 5). Under mainly easterly wind conditions (Fig. 7), offshore waters were advected towards the coast, where the SSH increased and subsequently sank in a downwelling-type Ekman circulation, similar to the findings of [45]. No significant vertical displacement of water was indicated in this period in the surface relative vorticity field (Fig. 11).

The later occurrence of the peak of the bloom at points PT2 and PT3 (Table 1) compared with the other points suggested that a fully developed winter mixed layer was necessary to ensure the entrainment of nutrients within the euphotic zone. Because upwelling is unlikely at these points due to their location, nutrient supplies most likely stem from recycling and resuspension. Because point PT3 is shallower and located closer to shore than the other points, the site is more susceptible to advective mixing caused by vertical current shear associated with changes in wind direction or intensity, which can occur in either season. This mixing may also be promoted by the downwelling-type Ekman circulation associated with southeast-east winds during the dry season. Moreover this point was subjected to positive relative vorticity throughout the year. Thus, the seasonal cycle of the mixed layer depth at PT3 was not as pronounced as that visualized at the other points and exhibited weaker coupling between the mixed layer depth and Chl-a than that observed at PT2.

## Conclusions

A combination of remote sensing data and numerical simulations was used to study the spatial and temporal variability of Chl-a and the effects of meteorological and oceanographic conditions on the modulation of this variability over the AB and RCB regions.

The system can be characterized by two distinct hydrodynamic patterns: stratified and mixed. During the wet season, a positive net heat flux favored the development of a seasonal thermocline. This thermocline stratified the water column, restricting the downward transference of wind stress momentum, reducing the mixed layer depth and reducing the recycling of nutrients, which restricted them primarily to the bottom. The stratification was enhanced by typical northerly and northeasterly upwelling-favorable winds, the negative relative vorticity induced in the onshore portion of the BC and the interaction of eddies and meanders with the shelf break that resulted in raising colder subsurface water onto the shelf.

With the onset of the dry season, the increase in latent heat loss and the reduction in shortwave radiation resulted in cooling of the surface layers. This cooling thus weakened the water column stratification, and drove the seasonal deepening of the mixed layer. This process was amplified by the southerly and southeasterly downwelling favorable winds that blew in the opposite direction to the main current system over the continental shelf. These winds induced vertical mixing by reverse shear, thus inhibiting re-stratification of the water column. Later in this season, easterly winds increased SSH close to the coast, which also resulted in downwelling-type Ekman circulation. It is in this period that Chl-a concentration started to increase, peaking between mid-May and the end of June, which characterizes the winter bloom in this region.

This study demonstrated that the seasonal phytoplankton bloom in the AB and RCB waters was strongly influenced by mixed layer depth. However, the phase and strength of the seasonal bloom presented spatial variability, influenced by different oceanographic conditions that affected water column stratification and nutrient supply. Whereas points PT1, PT4 and PT5 were more exposed to deep ocean forcing, such as input of momentum and properties induced by the eddies and meanders of the BC, points PT2 and PT3 were located in a more sheltered position and were subjected to shallow continental shelf dynamics (such as advective mixing and resuspension). Biological factors such as grazing, which were not considered this study, may also be important.

The main limitation of this study is to rely only on model simulations to assess the oceanographic and meteorological conditions in the region of study. However, the different model results present a consistent physical context, including robust explanations for the spatial and temporal structure of the phytoplankton seasonal bloom. Future observational programs are required to circumvent the inadequacy of the oceanographic and meteorological data and confirm the findings of the present work.
